# Supplementary material for: Effects of more natural housing conditions on the muscular and skeletal characteristics of female C57BL/6J mice
Source: Lab Anim Res. 2023 May 16;39:9. doi: 10.1186/s42826-023-00160-9 (PMC10186785; doi:10.1186/s42826-023-00160-9)
Supplement: Supplementary file 1 — Additional file 1: Fig. S1. Structural femur properties of female C57BL/6J mice in three different housing conditions. A cortical porosity in % with respective p values from post hoc Wilcoxon test. B femur trabecular thickness in mm with respective p values from post hoc Tukey test. C number of femur trabecular bones in mm-1 with respective p values from post hoc Tukey test. D separation between femur trabecular bones in mm with respective p values from post hoc Tukey test. Table S1. Summarized results of examined parameters in addition to 3.3 Bone density and structural properties data and Table 1. Shown is the age of the animals at the respective time of measurement and the value of the parameter for the animals from the three housing conditions CON, ENR and SNE housing. Values are shown as the mean with the standard deviation (SD) and the coefficient of variance (CV). The housing condition showing the lowest CV is marked in the CV column in respective to the used color scheme (CON black, SNE green). [file 42826_2023_160_MOESM1_ESM.docx]

**Supplements**

Additional data to – 3.3 Bone density and structural properties data is provided in Figure S1 and Table S1

**
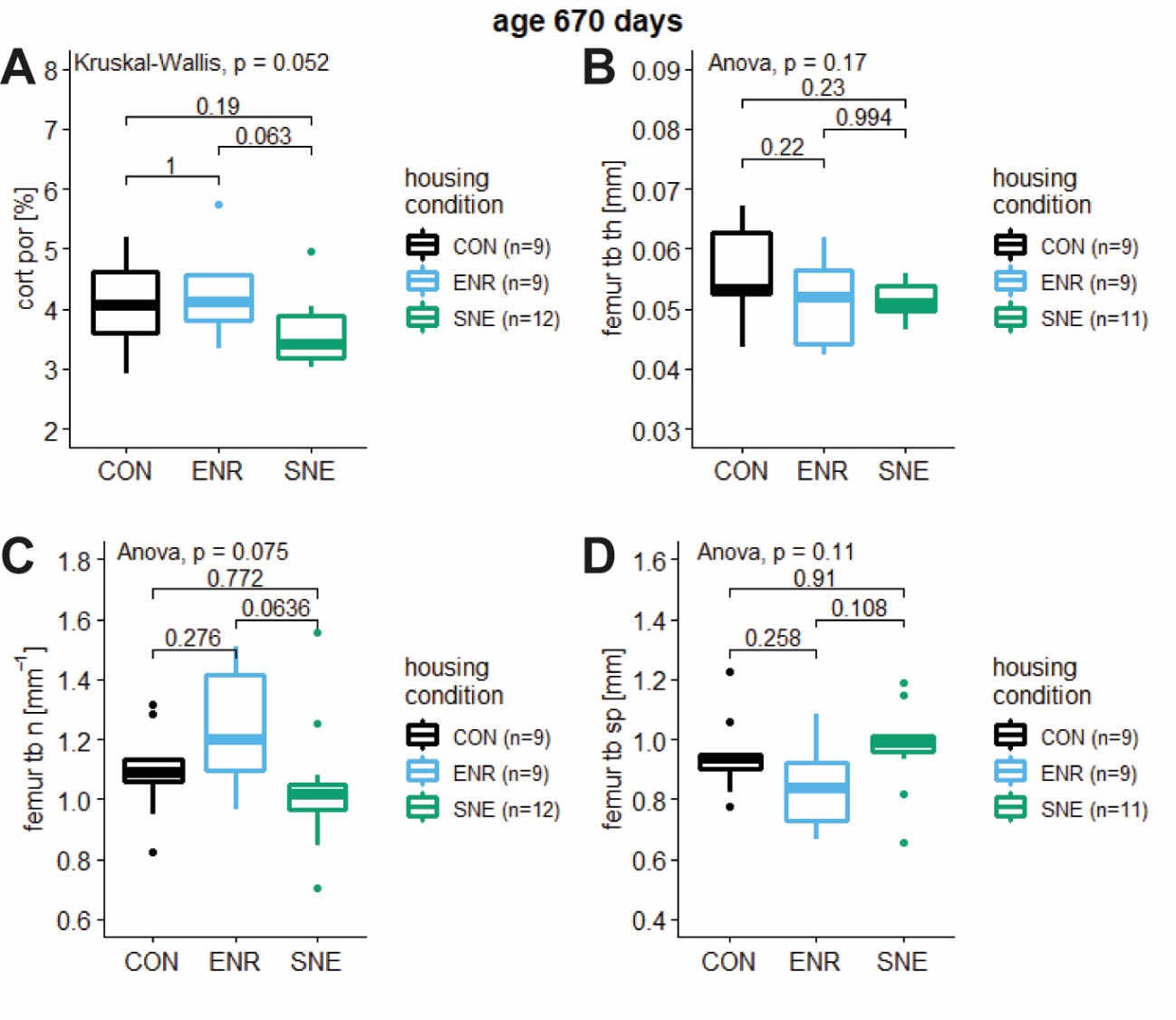
**

**Figure S1. Structural femur properties of female C57BL/6J mice in three different housing conditions.** A – cortical porosity in % with respective *p* values from post hoc Wilcoxon test. B – femur trabecular thickness in mm with respective *p* values from post hoc Tukey test. C – number of femur trabecular bones in mm^-1^ with respective *p* values from post hoc Tukey test. D – separation between femur trabecular bones in mm with respective *p* values from post hoc Tukey test.

**Table S1. Summarized results of examined parameters** in addition to 3.3 Bone density and structural properties data and ***Table 1.*** Shown is the age of the animals at the respective time of measurement and the value of the parameter for the animals from the three housing conditions CON, ENR and SNE housing. Values are shown as the mean with the standard deviation (SD) and the coefficient of variance (CV). The housing condition showing the lowest CV is marked in the CV column in respective to the used color scheme (CON black, SNE green).

| **animal age** | **parameter** | **housing condition** | **mean ± SD (max-min, n)** | **CV** |
| --- | --- | --- | --- | --- |
| days |  |  |  | % |
| 670 | cortical porosity (***Figure S1 A***) | CON | 4.1 ± 0.7 % (5.2 – 2.9 %, n = 9) | 17.6 |
|  |  | ENR | 4.2 ± 0.7 % (5.8 – 3.3 %, n = 9) | 16.7 |
|  |  | SNE | 3.6 ± 0.6 % (5.0 – 3-0 %, n = 12) | 15.5 |
|  | femur trabecular thickness (***Figure S1 B***) | CON | 0.056 ± 0.007 mm (0.067 – 0.044 mm, n = 9) | 13.2 |
|  |  | ENR | 0.051 ± 0.007 mm (0.062 – 0.042 mm, n = 9) | 14.3 |
|  |  | SNE | 0.051 ± 0.003 mm (0.056 – 0.046 mm, n = 11) | 6.4 |
|  | femur trabecular number (***Figure S1 C***) | CON | 1.09 ± 0.15 mm^-1^ (1.31 – 0.83 mm^-1^, n = 9) | 13.8 |
|  |  | ENR | 1.23 ± 0.20 mm^-1^ (1.51 – 0.97 mm^-1^, n = 9) | 16.0 |
|  |  | SNE | 1.03 ± 0.21 mm^-1^ (1.55 – 0.71 mm^-1^, n = 12) | 20.3 |
|  | femur trabecular separation (***Figure S1 D***) | CON | 0.948 ± 0.131 mm (1.224 – 0.777 mm, n = 9) | 13.8 |
|  |  | ENR | 0.843 ± 0.140 mm ( 1.085 – 0.668 mm, n = 9) | 16.6 |
|  |  | SNE | 0.974 ± 0.144 mm (1.187 – 0.654 mm, n = 11) | 14.8 |
